# Optimization of training for professional rugby union players: investigating the impact of different small-sided games models on GPS-derived performance metrics

**DOI:** 10.3389/fphys.2024.1339137

**Published:** 2024-02-12

**Authors:** Xiangyu Ren, Mathieu Henry, Simon Boisbluche, Kilian Philippe, Mathieu Demy, Shuzhe Ding, Jacques Prioux

**Affiliations:** ^1^ Sino-French Joint Research Center of Sport Science, Key Laboratory of Adolescent Health Assessment and Exercise Intervention of Ministry of Education, College of Physical Education and Health, East China Normal University, Shanghai, China; ^2^ Movement, Sport, Health Laboratory, Rennes 2 University, Bruz, France; ^3^ Department of Sports Sciences and Physical Education, École Normale Supérieure de Rennes, Bruz, France; ^4^ Rugby Club Vannes, French Rugby Federation, Vannes, France; ^5^ Laboratory of Movement, Balance, Performance and Health (MEPS, EA-4445), University of Pau and Pays de l’Adour, Tarbes, France

**Keywords:** constraints-led approach, external load, global positioning system, team sports, intermittent exercise

## Abstract

**Introduction:** Professional rugby union players can improve their performance by engaging in small-sided games (SSGs), which simulate the movement patterns of the game. This study collected metrics related to running performance and mechanical workload and their relative values from both forward and back positions, aiming to explore the impact of different SSGs factors on athlete workload, as well as the workload difference between official games (OGs) and SSGs.

**Methods:** The monitored GPS data were collected from SSGs with different player numbers and pitch sizes (five sessions), SSG rules (5 weeks, four sessions per week), and OGs conducted throughout the year. Additionally, the study compared changes in players’ sprinting performance before and after two SSG sessions.

**Results:** Backs had greater workload than forwards. Less space and number of players SSG (4 vs. 4, 660 m^2^) was conducive to facilitating training for players in acceleration and deceleration. Conversely, larger spaces were associated with improved running performance. However, the introduction of a floater had no significant impact on performance improvement. Additionally, the 7 vs. 4 model (seven players engaged with four opponents) resulted in the greatest workload during medium-hard accelerations (F = 52.76–88.23, *p* < 0.001, η_p_^2^ = 0.19–0.28). Japan touch model allowed for more high-speed running training (F = 47.93–243.55, *p* < 0.001, η_p_^2^ = 1.52). The workload performed by SSGs can almost cover that of OGs (F = 23.36–454.21, *p* < 0.05, η_p_^2^ = 0.03–0.57). In the context of η_p_^2^, values around 0.01, 0.06 and 0.14 indicate small, medium and large effects respectively.

**Discussion:** However, given the significantly higher workload of SSGs and the slight decrease in sprinting performance, further research is required to examine the training patterns of SSGs. This study provided insight into the impact of player numbers, pitch size, and rules on rugby-specific SSGs. Coaches should optimize SSG setups for enhanced training outcomes, ensuring the long-term development of physical capacity, technical and tactical skills.

## Introduction

Rugby union (RU) is a dynamic, field-based team sport that combines high-intensity (collisions, accelerations, and changes of direction) with low-intensity (jogging and walking) activity (Duthie et al., 2003a). This multifaceted movement requires players to detain a vast and varied skill set. It is therefore essential that the training strategy is optimized and organized to focus on the development of technical (tackling, rucks, mauls) and tactical (game situation adaptability) skills. Moreover, the development of physical attributes such as maximal strength, power, cardiovascular capacity and tendomuscular robustness is a crucial aspect of a RU player’s preparation. These attributes greatly influence a team’s performance as they engage in various running activities throughout the game (Gabbett et al., 2007). Repeated high-intensity and skill exercises are typical training methods in RU, but they are insufficient to satisfy the demands of the professional setting. To date, strength and endurance training rarely expose athletes to on-field situations. This omission may hinder the players’ opportunity to develop decision-making skills in a dynamic environment (Gabbett et al., 2012; Davids et al., 2013), which is essential to achieve gameplay performance outcomes. In RU training where time dedicated to physical enhancement is scarce, the necessity for concurrent training methods has led to the inclusion of small-sided games (SSGs).

Over the last 20 years, coaches have adopted SSGs as a training method for team sports (Gamble, 2004; Gabbett, 2006; Dellal et al., 2011a). Indeed, SSGs provide a dynamic environment that is simple to adapt to, requiring fewer players and smaller pitch sizes than traditional games. The aim is to create sub-environments that imitate the stress and fatigue players experience during games, while developing an athlete’s stamina, muscular strength, mental fortitude, and game-play abilities (Gabbett et al., 2009). Coaches can manipulate the impact of SSGs on players’ physiological and perceptual responses.

Workload quantification is essential for a more profound comprehension of the dose-response relationship between stress and internal responses. It requires meticulously recording the demands of both training and competition (Meir et al., 1993; Bourdon et al., 2017; Impellizzeri et al., 2023). This quantification can manifest as external load, representing the work completed by an athlete independently of their internal characteristics (Wallace et al., 2009), or internal load, encompassing all psychophysiological responses occurring during the execution of exercise prescribed by the coach (Impellizzeri et al., 2019). To date, many research investigations have analyzed the workload of different SSG models by manipulating pitch size, player density, rules, and other variables. Specifically, most studies have demonstrated that larger playing pitch size was correlated with increased heart rate (HR) (Atlı et al., 2013), lactate concentration and subjective ratings of perceived exertion (RPE) (Kennett et al., 2012b). Furthermore, reducing the amount of participating players could raise HR reserves (Dellal et al., 2011b). Recent advancements in global positioning systems (GPS) technology have made it possible to obtain valid and reliable assessments of external load (Teixeira et al., 2021; Clavel et al., 2022; Crang et al., 2022). In this regard, recent studies in soccer and rugby have indicated that the SSG models involving reduced player numbers or larger fields led to the highest time-motion variables (Hill-Haas et al., 2010; Kennett et al., 2012a). In addition, when the rules changed (e.g., the number of ball contacts allowed was reduced), the high-speed running distance (HSR) increased (Castellano et al., 2013).

When utilizing GPS data collection, the primary focus often centers around speed zone distances, with a particular emphasis on HSR as crucial metrics for performance assessment. Total distance (TD) is the second most commonly captured metric, followed by sprints and meters per minute (West et al., 2019). These parameters are closely associated with the skill level of athletes and the scores in match play (Dalton-Barron et al., 2020). Players with excellent repeated sprinting abilities demonstrate higher rates of running at speeds greater than 5 m.s^−1^ per minute in match play (7.9 ± 1.0 m.min^−1^). Players with long-duration and high-intensity intermittent running capabilities exhibit longer TD covered (6,800 ± 1,969 m), with distances for speeds between 0 and 5 m·s^−1^ (6,309 ± 1,582 m) and exceeding 5 m.s^−1^ (490 ± 141 m) being greater (Gabbett et al., 2013). Among winning teams, displacement variables are notably high, encompassing TD, low-speed running distance, acceleration (Gabbett, 2013) and decelerations times (Kempton et al., 2017). The purpose of the acceleration-based external load indicator therein is to provide an estimate of whole-body mechanical load (Hollville et al., 2021) (i.e., external forces applied to the body/biomechanical loading experienced by the musculoskeletal system) (Vanrenterghem et al., 2017). In addition to fundamental motion analysis measurements, player load (PL) is an index based on acceleration measurements that can be effectively utilized to quantify running demands (Roe et al., 2016), with relative values reaching 7.2 to 10.4 (SD: 0.8–2.0) during the match (Gabbett, 2015). Repeated high-intensity effort (RHIE), as a composite matric of contact, acceleration, or sprint, were associated with higher HR and perceived exertion (Johnston and Gabbett, 2011), occurring in proximity to key events (11 ± 6) (Gabbett and Gahan, 2016; Sheehan et al., 2022). Relevant professionals can utilize the above information to devise strategies for physical training of sufficient intensity and implement recovery protocols.

However, it might be challenging for coaches to plan the optimal training framework and to manage the exercise’s overall intensity as physical and technical demands are highly sensitive when SSGs settings change (Dellal et al., 2008; Owen et al., 2012). Additionally, poorly designed drills, such as those with inappropriately sized width, too many participants or inappropriate rules, can also have detrimental effects by raising the possibility of contact injuries (Clemente, 2020). To address these limitations, training goals must be accurately and thoroughly established when SSG sessions are designed and implemented. Moreover, there is a lack of comparative research between the external load of SSGs and official games (OGs) in RU. Hence, more research is required to establish the validity of these training methods and to explore their potential in provoking specific physiological, technical, and tactical adaptations.

The aims of this study were twofold, first we wanted to quantify performance outcome by investigating the effects of different numbers of players (4 vs.4 to 8 vs. 8), pitch sizes (660, 900, 1,080, 2,500 m^2^), and rules during SSGs. Secondly, we aimed to describe and compare the external load of OGs and SSG models in relation to their goals. Given that a RU player’s physical demands differ according to their position (Darrall-Jones et al., 2015), it is critical to analyze these effects on the forwards and backs groups as independent populations. Thus, we hypothesized that changing different settings in SSGs would result in varying workload differences among forwards and backs (Dudley et al., 2023), and that the physical demands of external load indicators in SSGs would fulfil the requirements observed during games (Sarmento et al., 2018).

## Methods

### Participants

Forty professional RU players (age: 25.07 ± 4.82 years; height 1.85 ± 0.09 m, with forwards 1.88 ± 0.09 m and backs 1.84 ± 0.09 m; body mass 102.48 ± 15.7 kg, with forwards 111.07 ± 14.79 kg and backs 90.29 ± 5.74 kg) from the same team (French second division rugby championship, Pro D2) participated voluntarily in the research. A minimal sample size was estimated *a priori* with G*Power software3.1.9.7 (University of Dusseldorf, Dusseldorf, Germany). The estimation was performed using a small-to-medium effect size (ES) f = 0.25, partial eta squared (η_p_^2^) = 0.06, α error prob = 0.05, power (1-β error prob) = 0.8, numerator df = 3. The result showed a suitable total sample size of 22 players for actual high power (80.03%). Before the start of the protocol, subjects attended a presentation to receive information outlining the experimental procedures. All players were provided informed consent, aligning with the principles outlined in the Declaration of Helsinki. They retained the freedom to discontinue their involvement in the study at any point without facing any adverse consequences. The execution of the study protocol received assistance from both the medical and technical personnel affiliated with the professional team. Finally, the study respected the ethical guidelines of Rennes University and the research laboratory associated with this study.

### Study design and settings

**FIGURE 1:**
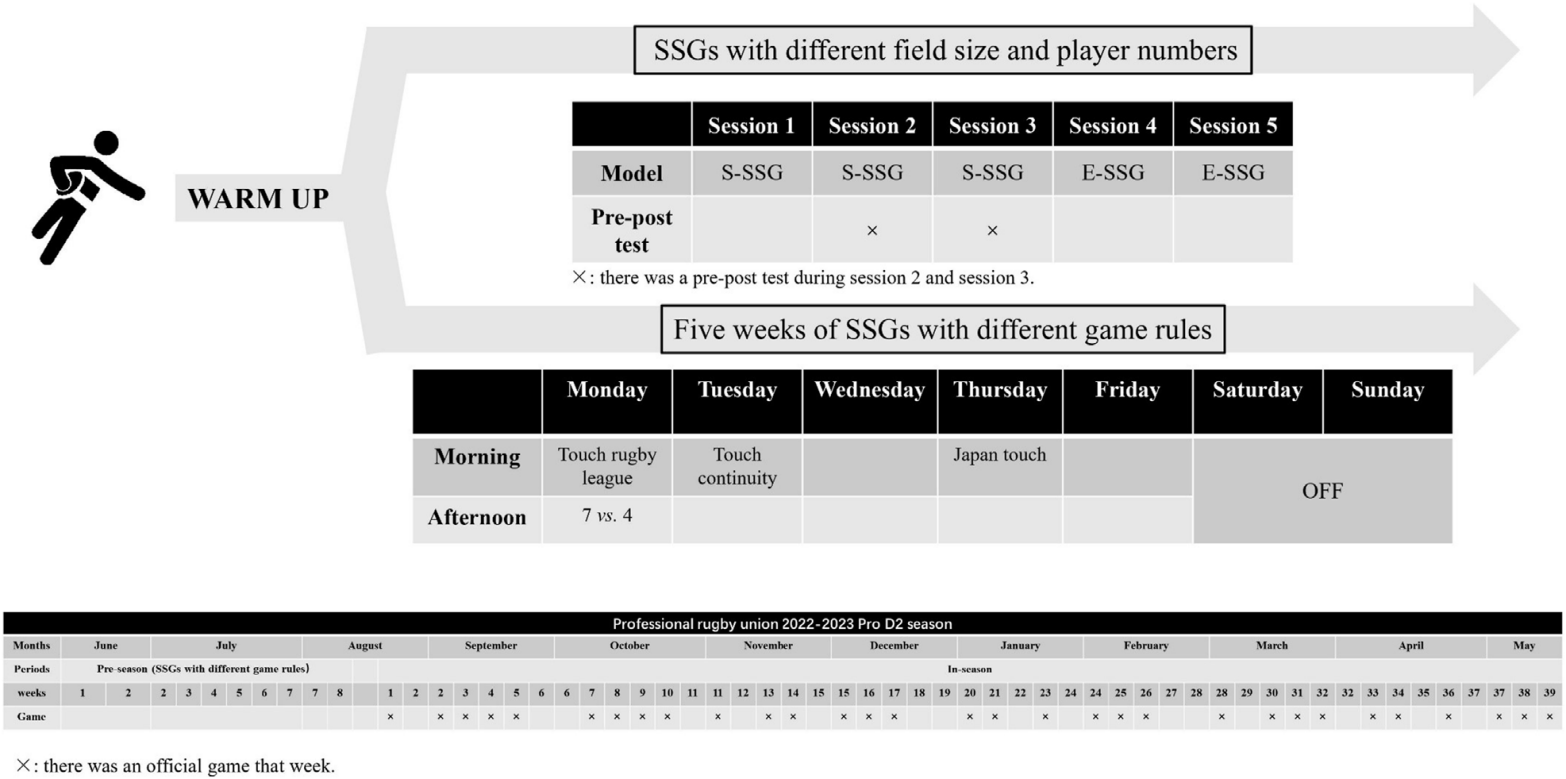
The arrangement of SSGs and organization of the 2022–2023 Pro D2 season.

This research was based on two main categories of SSG models that investigate the impact of modifying pitch size, player numbers and different game rules on external workload in RU. Data collection occurred during the 31 OGs in an entire season. 7 days separated each game (Figure 1). All SSG models and OGs were conducted on the same field and under similar temperature and relative humidity (Varley et al., 2012). All SSGs assessment protocols were completed after a 15-minute warm-up (i.e., dynamic stretching, mobility, and muscle activation). The coach supervised and ensured, through verbal encouragement, that athletes provided maximal exertion. During the study phase, every player was required to standardize their caloric intake and hydration status at least 24 h before each test day.

### Procedures

#### Performance monitoring

The application of GPS devices, for quantifying physical attributes (the player’s speed, acceleration, distance covered, etc.) in team sports, demonstrates remarkable effectiveness and reliability (Houy, 2020). GPS signals provide information about speed, distance, position, and acceleration of player movements during drills and official matches. Training sessions on different SSG models were recorded using GPS and Global Navigation Satellite System (GLONASS) technology (Vector Stadium Receiver, Optimeye X7 sensors, Catapult Sports^®^, Australia). When compared to cells sampled at lower frequencies, the 10 Hz cell produces the most efficient and reliable data (Akenhead et al., 2014). The GPS unit also includes a tri-axial accelerometer and gyroscope sampled at 100 Hz to provide higher velocity and acceleration accuracy, physical collision, and RHIE data. Each GPS sensor is equipped with a stretch vest that all players wear between their shoulder blades. These devices were activated 30 min before each training session to ensure a clear satellite reception. The GPS data were exported by applying specialist software (Openfield Console 3.7) for subsequent analysis.

#### 10-m and 20-m sprint test

Given that RU typically involves short sprints of 10 m with player’ average sprint distance ranging between 15 and 21 m (Gabbett, 2012), we conducted 10-m and 20-m sprint test. These tests were employed both before and after two training sessions using S-SSG model to assess each player’s acceleration ability and their performance changes.

Sprint speed was assessed by 10- and 20-m sprint times using dual beam electronic timing gates (Swift Performance Equipment, New South Wales, Australia). All running tests were conducted on a rugby pitch (natural turf). After a standardized warm-up, players performed two sprint trials interspersed with 1-min rest periods. Light gates were positioned at the 10 m and 20 m marks to evaluate the time taken to reach each distance. All players started with the front foot positioned 0.5 m behind the starting line, and players were instructed to run as fast as possible for a distance of 20 m from standing. The best score for each distance was recorded as the test score (Gabbett et al., 2008; Comfort et al., 2012; Zabaloy et al., 2021). The within-trial validity and reliability of the above procedure have been established (Chiwaridzo et al., 2017).

### Task design of SSGs

#### Effects of modifying pitch size and player numbers

SSG models included free play with a focus on ball possession. In this category of models, SSGs played on strength training days were described as strength SSG (S-SSG) models, whereas SSGs performed on endurance training days were referred as endurance SSG (E-SSG) models. The SSGs training protocols were specially prescribed and implemented by the team coaches. In the framework of the team’s tactical approach, players were involved in five SSG models: Three S-SSG models (4 vs. 4, 4 vs. 5, and 5 vs. 5) and two E-SSG models (7 vs. 7, and 8 vs. 8) (Table 1). Where 4 vs. 5 employs the 4 vs. 4 + 1 floater format, which essentially represented the coach’s attempt to introduce a variant based on the 4 vs. 4 SSG. In this session, both the pitch size and the rules of the SSG remained consistent with the 4 vs. 4 SSG.

**TABLE 1:** Training organization of five small-sided games models in eight sessions.

`	Number of players/bouts	Number of bouts	Bout duration (min)	Rest interval between the bouts (min)	Pitch size (m^2^)	Relative pitch size (m^2^)
Session 1	4 vs. 4	3	1.5	1	660	82.5
Session 2	4 vs. 5 (4 + 1 floater)	3	1.5	1	900	100
Session 3	5 vs. 5	3	1.5	1	1,080	108
Session 4	7 vs. 7	3	3	1	2,500	170
Session 5	8 vs. 8	3	3	1	2,500	156

S-SSG, SSGs played on strength training days; E-SSG, SSGs performed on endurance training days.

Floater: A player who has a flexible or floating role within the team. This player is not restricted to a specific position on the field and may move around as needed during the game. The floater adapts to the dynamic situations on the field and can contribute to various aspects of the game, such as attack, defense, or transitions between the two.

#### Effects of modifying game rules

The SSG models were structured around four distinct rules (Table 2). With the aim of enhancing endurance and acceleration, touch rugby league (TRL) sessions were conducted on Monday mornings. 7 vs. 4 sessions were employed on Monday afternoons to target small-space acceleration. Touch continuity (TC) sessions took place on Tuesday mornings to achieve high intensity. Japan touch (JT) sessions were held on Thursday mornings with the purpose of emphasizing speed. The changes in pitch size and number of players in 7 vs.4 SSGs were made within the constraints of the rules. There were inherent correlations between these changes, and therefore they can be considered as a whole for comparison with SSGs of other rules. The four models were carried out over a period of 5 weeks, with four training sessions per week.

**TABLE 2:** Small-sided games models with four sets of rules.

	Touch rugby league	7 vs. 4	Touch continuity	Japan touch
Number of players/bouts	8 vs. 8	7 vs. 4	8 vs. 8	8 vs. 8
Number of bouts	3, 4 or 5	4	3, 4 or 5	3, 4 or 5
Pitch size (m^2^)	2,800	400	2,800	2,800
Bout duration (min)	1.5–2.75	1.5	1.5–2.75	1.5–2.5
Rest interval between the bouts (min)	1.25	1.25	1.25	1.25
Scoring	Flatten in-goal	Flatten in-goal	Flatten in-goal	Flatten in-goal
Restart of play after a try	Direct for the same team	Direct for the same team	Direct for the same team	Direct for the same team
Kicking	Yes (1/bouts)	No	Yes (1/bouts)	Yes (1/bouts)
Contact	2-hand touch	2-hand touch	2-hand touch	2-hand touch
Number of touches	4	1	3	2
Objective	Aerobic	Strength	Between aerobic and speed	Speed
Rules	✧For first two touches, the defenders go back to the 5 m behind the ruck	✧The attackers score after the 5 m line without touch	✧Prior to or during the tackle, or once on the ground, the obligation to keep the ball alive through an axial pass	✧Each team consists of four players
	✧The attackers have 3 seconds to release the ball from any ruck	✧Score try: put the ball after 5 m	✧When the defender touches a player, he has to sprint in his camp and go back	✧Play with two corners
			✧After each touch, there is one defender less	✧If a ruck occurs, the attacking team with eight players engages while the defensive team (four players) needs to sprint and touch the line before they can defend again
				✧One ruck opportunity to play 8 vs. 4 to score

### Study variables

The metrics of running capability were reported as TD covered, maximum running velocity (V_max_), HSR (>15 km.h^−1^) (Waldron et al., 2011; Kempton et al., 2014), very high-speed running (VHSR) (>21 km.h^−1^) (Waldron et al., 2011; Kempton et al., 2014), and sprint running (SR) (>25 km.h^−1^) (Dubois et al., 2017; Vachon et al., 2022) distance. Mechanical workload metrics were reported as PL (arbitrary unit, AU), the number of medium acceleration (MA) (>2 m.s^−2^), distance of medium acceleration (D-MA) (>2 m.s^−2^), the number of hard acceleration (HA) (>2.5 m.s^−2^), distance of hard acceleration (D-HA) (>2.5 m.s^−2^), the number of medium deceleration (MD) (>2 m.s^−2^), the number of hard deceleration (HD) (>2.5 m.s^−2^) and RHIE. These metrics were expressed in absolute (ball in play) values. To allow for comparison between SSGs and OGs, these metrics were reported as per minute values.

### Statistical analysis

A total of 900 SSG data points were evaluated during the S-SSG and E-SSG training sessions. The number of participating players in S-SSG models was twenty-seven, with fourteen for forwards and thirteen for backs. E-SSG models involved thirty-three participating players, including sixteen for forwards and seventeen for backs. The SSGs with the rules of TRL, 7 vs. 4, TC, and JT were examined on a total of 9604 SSG data points. To describe the workload requirements of SSG models and OGs, a descriptive analysis was performed using the data as mean and standard deviation (mean ± SD). The coefficient of variation (CV) for SSG models was calculated to determine the variability of performance indicators. Before initiating the analysis of variance, we used the Shapiro-Wilk test and Levene’s test to determine the normality and homogeneity of variance in all of the data. When the data exhibited normal distribution and homogeneity of variance, Student’s t-test was employed to compare the differences between S-SSG model and E-SSG model. Considering the varying training participants and pitch sizes, this study employed a two-way analysis of variance (ANOVA) to investigate the interactions among these factors. A least significant difference (LSD) *post hoc* test was performed after significant main effects and factor interactions (Perneger, 1998). The paired samples *t*-test is employed to compare the differences in 10 m and 20 m sprint performance before and after training sessions using four S-SSG models. The Mann-Whitney U test, Kruskal-Wallis H-test and its subsequent *post hoc* comparison procedure (Dunn’s *post hoc* analysis) were applied due to the non-normal distribution of the GPS metrics, the heterogeneity of the variance and the comparison of SSG models and OGs. The statistical significance was set at *p* < 0.05. ES was evaluated using Cohen’s d and partial eta squared (η_p_^2^) along with a 95% confidence interval. Cohen’s d = 0.2, 0.5, and 0.8, η_p_^2^ = 0.01, 0.06 and 0.14 correspond to small, medium and large effects respectively (Cohen, 1992).

## Results

### Effects of modifying pitch size and player numbers

**FIGURE 2:**
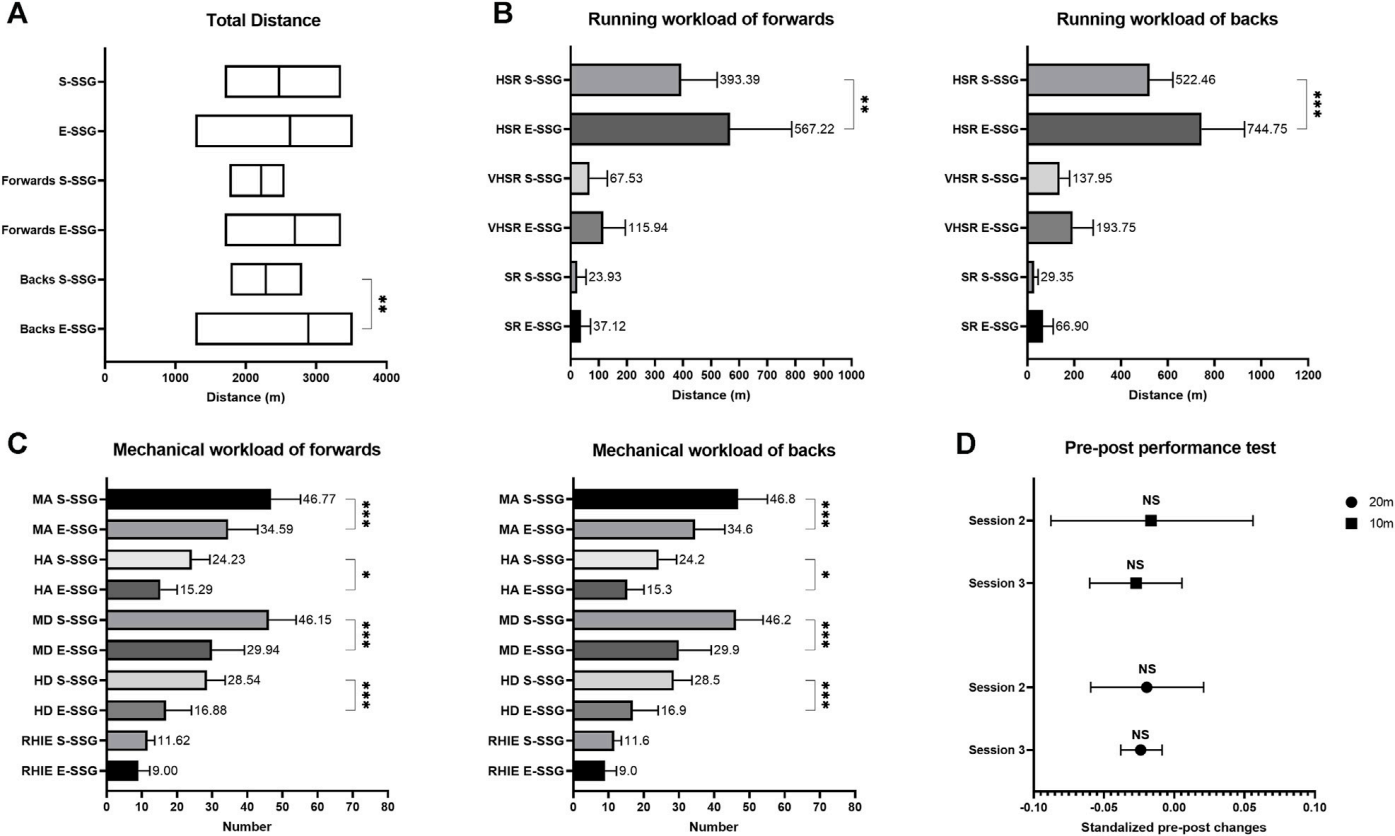
Comparison of workload and performance changes in S-SSG and E-SSG models. **(A)** Comparison of TD for forwards and backs. **(B)** Running workload comparison for forwards and backs. **(C)** Mechanical workload comparison for forwards and backs. **(D)** Difference between pre-and post-performance test on neuromuscular function in 10 m and 20 m. S-SSG, small-sided game played on strength training day; E-SSG, small-sided game performed on endurance training day; HSR, high-speed running (>15 km.h^−1^); VHSR, very high-speed running (>21 km.h^−1^); SR, sprint running (>25 km.h^−1^); MA, the number of medium accelerations (>2 m.s^−2^); HA, the number of hard accelerations (>2.5 m.s^−2^); MD, the number of medium decelerations (>2 m.s^−2^); HD, the number of hard decelerations (>2.5 m.s^−2^); RHIE, repetitive high-intensity exercise. *p < 0.05, **p < 0.01, ***p < 0.001.

High variability was observed in the metrics of VHSR (CV = 0.63) and SR (CV = 1.10). Conversely, mechanical workload and TD exhibited low variability (CV = 0.11–0.41) (Table 3). When evaluating the running performance, our results show that the TD of backs and HSR of both positions in E-SSG models outperformed S-SSG models (Figures 2A, B, *p* < 0.01, ES = 1.52, 1.14 and 1.58). This trend was further supported by the statistical results presented in Table 3, where the *p*-values and Cohen’s d effect sizes highlighted significant differences. For example, the ES for the absolute value of TD was −1.3, and it was −1.13 for HSR. Similarly, with respect to mechanical workload, S-SSG models usually outperformed higher values compared to E-SSG models (Figure 2C, *p* < 0.05, ES = 0.01–0.91). These trends were also corroborated by the results presented in Table 3, where the corresponding *p*-values and Cohen’s d values emphasized substantial effect sizes. The relative values of MA+MD and HA+HD were specifically noticeable, with effect sizes of 1.33 and 1.41, respectively. Figure 2D illustrates the differences between the pre-performance and post-performance tests on neuromuscular function, and the results show no significant difference in sprint performance.

**TABLE 3:** Mean ± SD values of metrics taken from S-SSG and E-SSG models.

		S-SSG models	CV	E-SSG models	CV	*p*-value	Cohen’s d
Subjects		52		47			
Ball in play (min)		15.9 ± 2.51	0.16	22.1 ± 4.15	0.19	<0.001	−1.8
TD	Absolute (m)	2,250.8 ± 253.2	0.11	2,795.1 ± 535.3	0.19	0.004	−1.30
	Relative (m.min^−1^)	145.4 ± 12.0	0.08	136.7 ± 10.6	0.08	<0.001	0.77
HSR	Absolute (m)	455.5 ± 130.8	0.29	658.7 ± 218	0.33	0.054	−1.13
	Relative (m.min^−1^)	33.5 ± 8.5	0.25	29.1 ± 8.9	0.31	0.015	0.51
VHSR	Absolute (m)	101.4 ± 63.9	0.63	152.5 ± 93.9	0.62	0.042	−0.64
	Relative (m.min^−1^)	8.2 ± 4.3	0.53	6 ± 3.6	0.59	0.012	0.53
SR	Absolute (m)	20.3 ± 22.4	1.10	42.8 ± 43.1	1.01	0.259	−0.65
	Relative (m.min^−1^)	2.0 ± 1.8	0.89	1.5 ± 1.7	1.17	<0.001	0.30
MA+MD (n)	Absolute	81.5 ± 18	0.22	58.1 ± 17.2	0.30	0.001	1.33
	Relative	4.6 ± 1.6	0.34	3.4 ± 1.2	0.35	<0.001	0.86
HA+HD (n)	Absolute	44.3 ± 12.9	0.29	27.3 ± 11.1	0.41	0.001	1.41
	Relative	2.5 ± 1	0.41	1.6 ± 0.8	0.50	<0.001	0.89

TD, total distance (m); HSR, high speed running (>15 km.h^−1^) distance (m); VHSR, very high-speed running (>21 km.h^−1^) distance (m); SR, sprint running (>25 km.h^−1^) distance (m); MA+MD, the number of mean accelerations+decelerations (>2 m.s^−2^); HA+HD, the number of high accelerations+decelerations (>2.5 m.s^−2^). Typical error of measurement expressed as the coefficient of variation (CV) (95% confidence limits).

**FIGURE 3:**
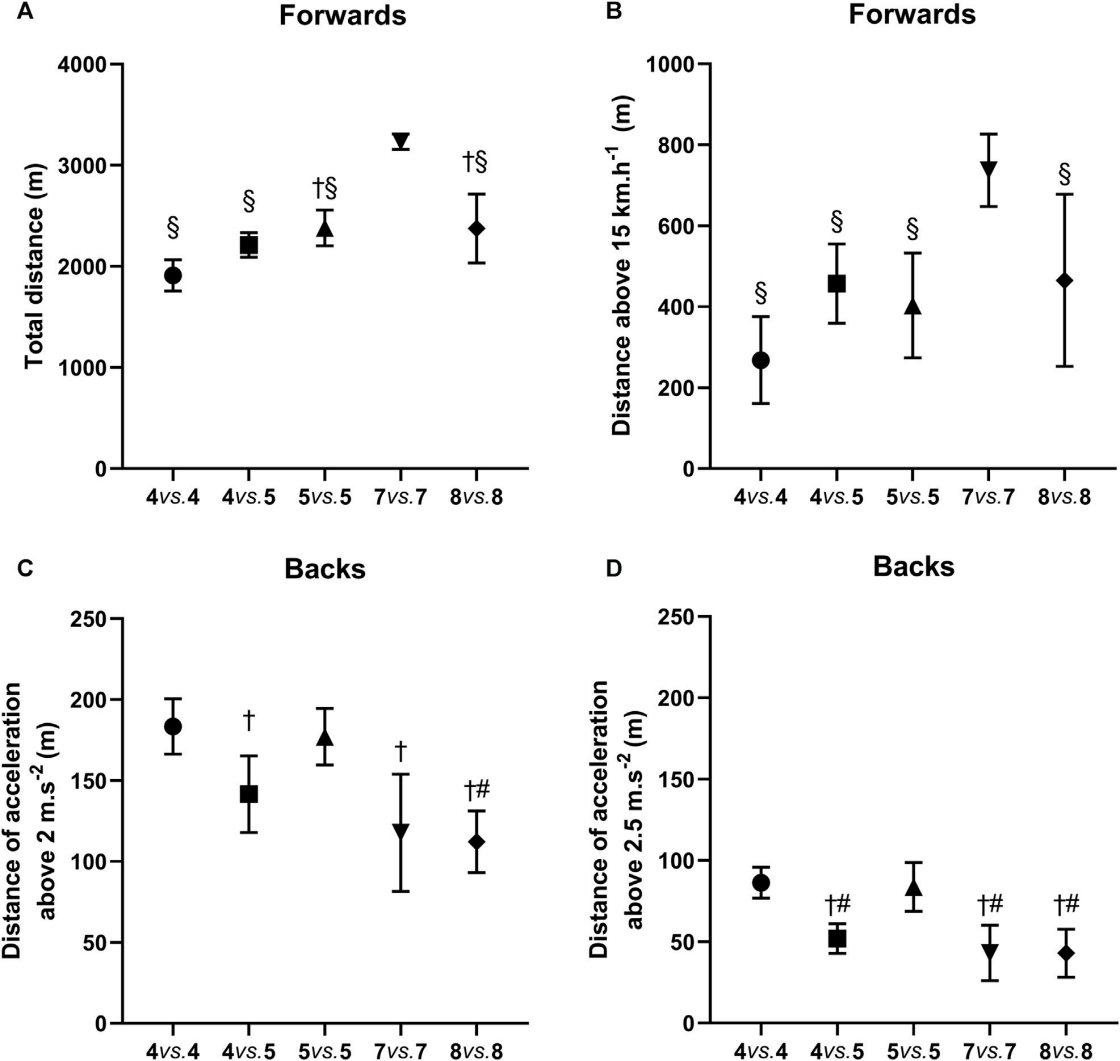
Effect of player numbers on performance in SSG models for forwards and backs. **(A)** Impact of the number of players on TD performance for forwards. **(B)** Impact of the number of players on HSR performance for forwards. **(C)** Impact of the number of players on D-MA performance for backs. **(D)** Impact of the number of players on D-HA performance for backs. §: Compared to 7 vs. 7, *p* < 0.05; †: Compared to 4 vs. 4, *p* < 0.05; #: Compared to 5 vs. 5, *p* < 0.05.

Figure 3 highlights the impact of varying player numbers within the SSG models on performance outcomes as a function of position. In Figures 3A, B, the TD and HSR covered by forwards was highest (*p* < 0.05, η_p_^2^ = 0.46 and 0.44) in the 7 vs. 7 model. Among backs, the 4 vs. 5 model demonstrate a notably lower (*p* < 0.05, ES = 0.95) D-MA performance compared to the 4 vs. 4 model (Figure 3C). Furthermore, the D-HA metric values within the 4 vs. 5 model were the lowest (*p* < 0.05, η_p_^2^ = 0.40) among all S-SSG models (Figure 3D).

### Effects of modifying game rules

The results provided in Table 5 present the distinctions among the four SSG models. In the 7 vs. 4 model, TD, V_max_, HSR, VHSR, SR, and PL showed the minimum values (F = 47.93–243.55, *p* < 0.001, η_p_^2^ = 0.17–0.52). On the contrary, players performed the best (F = 52.76–88.23, *p* < 0.001, η_p_^2^ = 0.19–0.28) in terms of both distance covered and times during acceleration and deceleration in this model. Maximum velocity, HSR and VSHR represented the biggest workload (F = 99.12–243.55, *p* < 0.05, η_p_^2^ = 0.22–0.52) than the other three SSG models during JT, while players in the TRL model had the lowest (F = 10.08, *p* < 0.05, η_p_^2^ = 0.04) RHIE. In the TC model, the player’s workload covered maximum TD and PL. Besides, differences (*p* < 0.05, ES = 1.3) in PL were observed between 7 vs. 4 and TRL models, as well as RHIE (*p* < 0.05, ES = 0.42) in the forwards and not in the backs. No significant differences were observed between TRL, TC, and JT models for acceleration and deceleration-related metrics (Table 5).

### Comparison between forwards and backs

In both the S-SSG and E-SSG models, the workload of the backs consistently remained higher (Table 4, *p* < 0.01) than that of the forwards, with the exception of TD. Meanwhile, as for the SSG models with four different rules, forwards had lower values (*p* < 0.01, ES = 0.17–0.54) compared to backs, except for TD in the 7 vs. 4, TC, and JT models and PL across all models. Also, the SR metric did not exhibit a significant difference (Table 5, *p* = 0.15, ES = 0.13) between forwards and backs in the 7 vs. 4 model.

**TABLE 4:** Comparison between forwards and backs workload (mean ± SD) in S-SSG and E-SSG models.

	S-SSG models				E-SSG models			
	Forwards	Backs	*p*-value	Cohen’s d	Forwards	Backs	*p*-value	Cohen’s d
TD	2,219 ± 233.4	2,285.1 ± 278.2	0.509	0.26	2,696.1 ± 505.5	2,888.2 ± 560.8	0.310	0.36
V_max_	24.8 ± 2.5	27.8 ± 1.7	0.001	1.43	26 ± 2.8	28.5 ± 2.7	0.013	0.91
HSR	393.4 ± 128.1	522.5 ± 99.5	0.008	1.13	567.2 ± 219.7	744.8 ± 183.1	0.017	0.88
VHSR	67.5 ± 62.8	138 ± 42.2	0.002	1.32	108.7 ± 81.2	193.7 ± 87.8	0.007	1.01
SR	12 ± 24.4	29.4 ± 16.6	0.042	0.83	25.5 ± 32.9	59 ± 46.2	0.023	0.84
MA+MD	70.9 ± 14.3	92.9 ± 14.3	0.001	1.54	51.3 ± 16.1	64.5 ± 16	0.025	0.82
HA+HD	36.4 ± 11.7	52.8 ± 8	<0.001	1.63	22.1 ± 9.8	32.2 ± 10.3	0.007	1.01
MA	36.1 ± 8.6	46.8 ± 8.4	0.003	1.25	27.6 ± 8.5	34.6 ± 8.4	0.023	0.83
HA	16.8 ± 7.5	24.2 ± 5.1	0.006	1.16	10.6 ± 4.4	15.3 ± 4.8	0.006	1.02
D-MA	128 ± 39	171.8 ± 24.4	0.002	1.35	88 ± 26.4	115.4 ± 29.7	0.009	0.98
D-HA	52.4 ± 23	77.5 ± 18	0.004	1.22	27.8 ± 10.7	43 ± 15.6	0.003	1.14
MD	34.8 ± 7.3	46.2 ± 7.7	0.001	1.51	23.8 ± 9.3	29.9 ± 9.2	0.065	0.67
HD	19.6 ± 6	28.5 ± 5.2	<0.001	1.59	11.4 ± 6.7	16.9 ± 7.2	0.032	0.78
RHIE	7.3 ± 3.1	11.6 ± 2	<0.001	1.66	5.1 ± 3	9 ± 3.3	0.001	1.23

TD, total distance (m); V_max_, maximum running velocity; HSR, high-speed running (>15 km.h^−1^) distance (m); VHSR, very high-speed running (>21 km.h^−1^) distance (m); SR, sprint running (>25 km.h^−1^) distance (m); MA, the number of medium accelerations (>2 m.s^−2^); MD, the number of medium decelerations (>2 m.s^−2^); HA, the number of hard accelerations (>2.5 m.s^−2^); HD, the number of hard decelerations (>2.5 m.s^−2^); D-MA, distance of medium acceleration (m); D-HA, distance of hard acceleration (m); RHIE, repetitive high-intensity exercise.

**TABLE 5:** Descriptive statistics (mean ± standard deviation) for the workload characteristics for SSG models with four rules.

Metric	TRL model		Comparison between positions		7 vs. 4 model		Comparison between positions		TC model		Comparison between positions		JT model		Comparison between positions	
	Forwards	Backs	*p*-value	ES	Forwards	Backs	*p*-value	ES	Forwards	Backs	*p*-value	ES	Forwards	Backs	*p*-value	ES
TD	1,167.9 ± 191.1^a^	1,282.2 ± 191.6^a^	<0.001	0.27	863.8 ± 181.3	834.6 ± 175.0	0.351	0.08	1,312.1 ± 230.6^b^ ^,^ ^c^ ^,^ ^d^	1,371.8 ± 217.0^b^ ^,^ ^d^	0.106	0.12	1,154.7 ± 146.3^e^	1,189.9 ± 156.1^e^	0.148	0.11
V_max_	23.7 ± 3.0^a^	26.1 ± 2.8^a^	<0.001	0.39	21.6 ± 1.9	22.9 ± 1.8	<0.001	0.32	24.1 ± 2.5^b^	25.9 ± 2.1^b^	<0.001	0.36	25.4 ± 2.6^e^ ^,^ ^d^ ^,^ ^f^	27.6 ± 2.5^e^ ^,^ ^d^ ^,^ ^f^	<0.001	0.39
HSR	191.6 ± 79.9^a^	287.0 ± 84.3^a^	<0.001	0.50	82.1 ± 39.9	108.4 ± 38.9	<0.001	0.33	260.9 ± 96.6^b^ ^,^ ^c^	355.7 ± 89.7^b^ ^,^ ^c^	<0.001	0.46	322.4 ± 89.3^e^ ^,^ ^d^ ^,^ ^f^	390.9 ± 83.4^e^ ^,^ ^f^	<0.001	0.37
VHSR	29.5 ± 30.6^a^	64.9 ± 40.8^a^	<0.001	0.47	6.4 ± 8.6	12.8 ± 10.5	<0.001	0.36	31.9 ± 26.9^b^	72.3 ± 36.2^b^	<0.001	0.54	63.2 ± 45.1^e^ ^,^ ^d^ ^,^ ^f^	111.6 ± 49.6^e^ ^,^ ^d^ ^,^ ^f^	<0.001	0.47
SR	6.2 ± 12.7^a^	15.4 ± 18.1^a^	<0.001	0.37	0.2 ± 1.0	0.6 ± 2.1	0.118	0.13	5.0 ± 8.8^b^	14.3 ± 17.0	<0.001	0.36	16.5 ± 24.0^e^ ^,^ ^d^ ^,^ ^f^	33.9 ± 31.7^e^ ^,^ ^d^ ^,^ ^f^	<0.001	0.34
MA+MD	29.7 ± 9.0	37.1 ± 10.1	<0.001	0.37	45.3 ± 13.1^b^ ^,^ ^a^ ^,^ ^e^	51.5 ± 14.1^b^ ^,^ ^a^ ^,^ ^e^	0.005	0.24	30.3 ± 7.6^d^	38.1 ± 9.0^d^	<0.001	0.42	27.7 ± 6.7	34.2 ± 7.5	<0.001	0.40
HA+HD	13.7 ± 5.8	18.9 ± 6.5	<0.001	0.37	23.8 ± 9.3^b^ ^,^ ^a^ ^,^ ^e^	29.6 ± 9.9^b^ ^,^ ^a^ ^,^ ^e^	0.001	0.29	14.6 ± 5.6	20.8 ± 6.8	<0.001	0.45	13.4 ± 4.3	19.0 ± 5.4	<0.001	0.50
MA	14.7 ± 4.9	18.1 ± 5.3	<0.001	0.30	22.5 ± 6.8^b^ ^,^ ^a^ ^,^ ^e^	25.8 ± 7.8^b^ ^,^ ^a^ ^,^ ^e^	0.011	0.22	15.1 ± 4.4	18.5 ± 5.9	<0.001	0.31	14.3 ± 3.4	17.4 ± 4.4	<0.001	0.35
HA	5.8 ± 2.8	8.0 ± 4.0	<0.001	0.28	10.2 ± 4.6^b^ ^,^ ^a^ ^,^ ^e^	13.3 ± 5.5^b^ ^,^ ^a^ ^,^ ^e^	0.001	0.29	6.4 ± 2.9	8.7 ± 4.5	<0.001	0.29	6.1 ± 2.3	8.4 ± 3.2	<0.001	0.35
D-MA	49.8 ± 17.2	63.2 ± 20.7	<0.001	0.32	76.6 ± 26.0^b^ ^,^ ^a^ ^,^ ^e^	90.9 ± 26.1^b^ ^,^ ^a^ ^,^ ^e^	0.001	0.27	48.9 ± 14.3	63.5 ± 22.7	<0.001	0.34	52.0 ± 14.6	68.7 ± 18.5	<0.001	0.44
D-HA	18.1 ± 9.3	24.9 ± 12.1	<0.001	0.29	332 ± 14.7^b^ ^,^ ^a^ ^,^ ^e^	43.6 ± 16.5^b^ ^,^ ^a^ ^,^ ^e^	<0.001	0.31	18.3 ± 7.7	26.3 ± 14.8	0.001	0.26	20.8 ± 9.3^d^	30.0 ± 13.3^d^	<0.001	0.35
HD	7.8 ± 3.7	10.9 ± 4.0	<0.001	0.37	13.6 ± 5.5^b^ ^,^ ^a^ ^,^ ^e^	16.3 ± 5.4^b^ ^,^ ^a^ ^,^ ^e^	0.004	0.24	8.2 ± 3.4	12.0 ± 3.5^d^	<0.001	0.50	7.2 ± 3.0	10.5 ± 3.6	<0.001	0.45
PL	129.9 ± 27.5^a^	140.0 ± 27.3^a^	0.011	0.17	101.8 ± 24.9	100.6 ± 21.9	0.835	0.02	146.9 ± 32.1^b^ ^,^ ^c^ ^,^ ^d^	148.3 ± 27.9^b^ ^,^ ^d^	0.561	0.04	127.7 ± 22.1^e^	129.3 ± 22.8^e^	0.647	0.04
RHIE	3.2 ± 1.8	5.2 ± 1.7	<0.001	0.48	4.0 ± 2.0^a^	5.4 ± 1.6	<0.001	0.35	4.1 ± 2.2^c^	6.1 ± 1.7^c^	<0.001	0.45	4.3 ± 1.9^f^	5.9 ± 1.4^f^	<0.001	0.42

TRL, Touch Rugby League; TC, Touch continuity; JT, Japan touch. TD, total distance (m); V_max_, maximum running velocity; HSR, high-speed running (>15 km.h^−1^) distance (m); VHSR, very high-speed running (>21 km.h^−1^) distance (m); SR, sprint running (>25 km.h^−1^) distance (m); MA, the number of medium accelerations (>2 m.s^−2^); MD, the number of medium decelerations (>2 m.s^−2^); HA, the number of hard accelerations (>2.5 m.s^−2^); HD, the number of hard decelerations (>2.5 m.s^−2^); D-MA, distance of medium acceleration (m); D-HA, distance of hard acceleration (m); PL, player load; RHIE, repetitive high-intensity exercise. ES, effect size.

^a^ 7 vs. 4 model vs. TRL model, *p* < 0.05.

^b^ 7 vs. 4 model vs. TC model, *p* < 0.05.

^c^ TC model vs. TRL model, *p* < 0.05.

^d^ TC model vs. JT model, *p* < 0.05.

^e^ 7 vs. 4 model vs. JT model, *p* < 0.05.

^f^ TRL model vs. JT model, *p* < 0.05. Players were in the same position when making comparisons. Corner markers were displayed where the value was high.

### Comparison between SSGs and OGs

**FIGURE 4:**
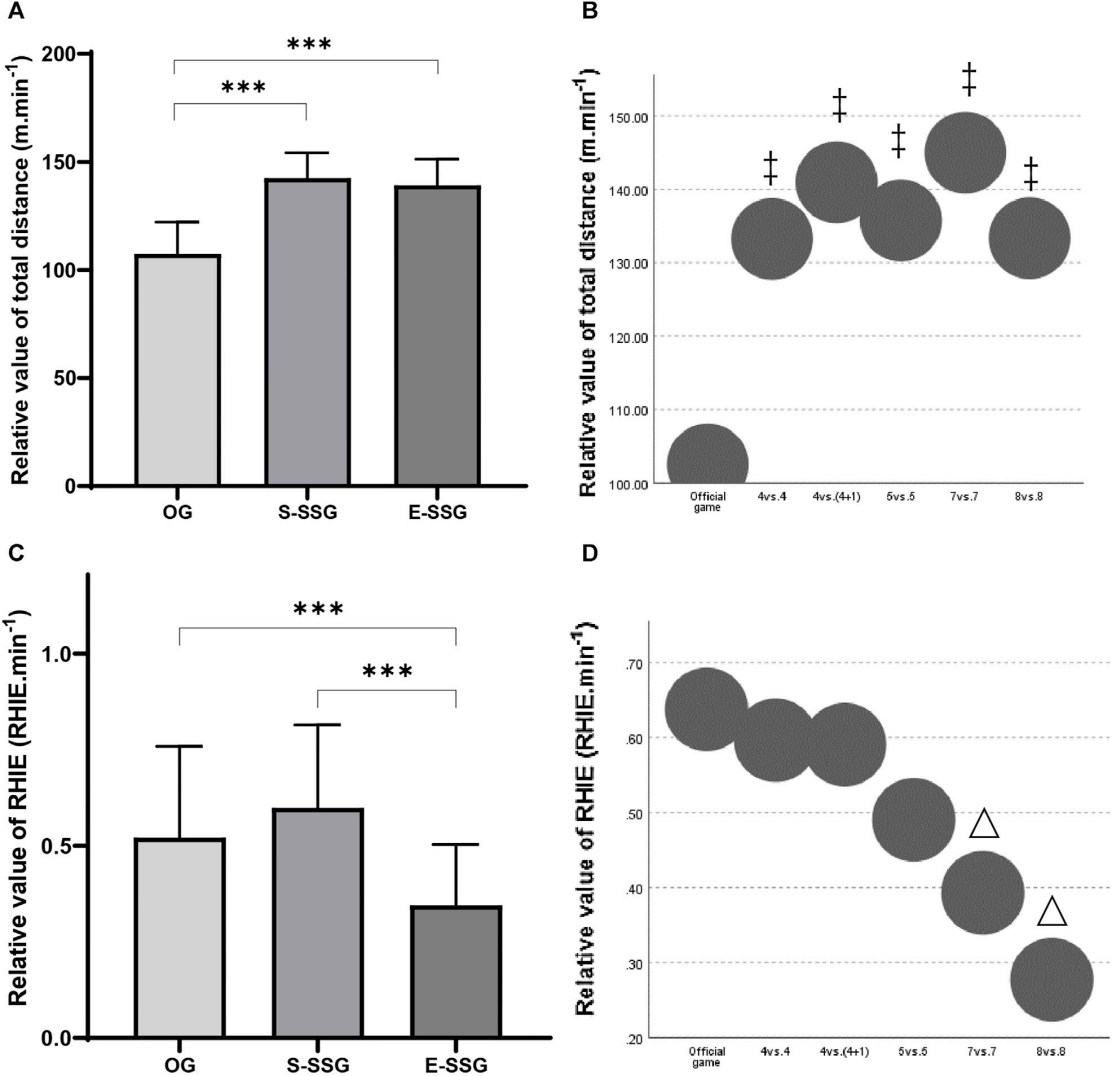
Comparison of relative performance metrics between SSG models and OGs. **(A)** Comparison of TD relative values (m.min^−1^) for S-SSG models, E-SSG models, and OGs. **(B)** Comparison of TD relative values (m.min^−1^) for different player numbers in SSG models and OGs. **(C)** Comparison of RHIE relative values (RHIE.min^−1^) for S-SSG, E-SSG, and OGs. **(D)** Comparison of RHIE relative values (RHIE.min^−1^) for different player numbers in SSG models and OGs. S-SSG, small-sided game played on strength training day; E-SSG, small-sided game performed on endurance training day; OG, official game. ‡: Compared to OG, *p* < 0.001; △: Compared to OG, *p* < 0.05.

In terms of OGs demand profiles, players had a relative distance (m.min^−1^) of 121.47 ± 22.08, a relative PL (PL.min^−1^) of 14.42 ± 2.71, and a relative RHIE (RHIE.min^−1^) of 0.54 ± 0.24. The relative workload of TD in S-SSG and E-SSG models exceeded the OGs requirements (Figures 4A, B, *p* < 0.001, ES = 1.45 and 1.44). However, E-SSGs did not cover the workload of OGs in RHIE.min^−1^ metrics (Figure 4C, *p* < 0.001, ES = 1.13). Besides, the 7 vs. 7 and 8 vs. 8 SSG models did not fulfill the RHIE.min^−1^ requirements of OGs (Figure 4D, *p* < 0.05, ES = 0.18 and 0.51).

**FIGURE 5:**
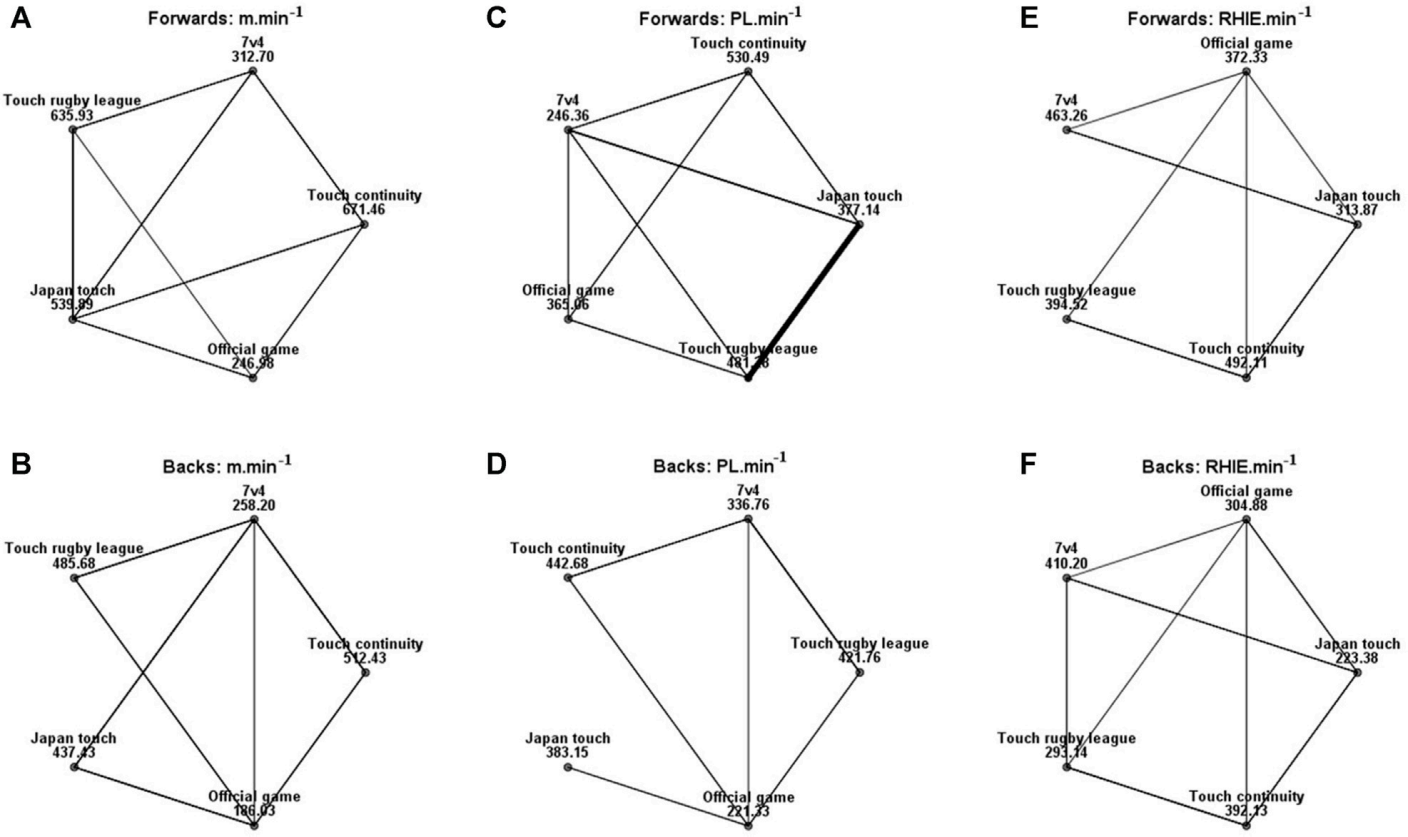
Pairwise comparison for forwards and backs in **(A, B)** m.min^−1^, **(C, D)** PL.min^−1^, and **(E, F)** RHIE.min^−1^ about SSGs and OGs analyzed. Each node showed the sample average rank of SSGs and OGs. The black line represented significant differences among groups (*p* < 0.05). m.min^−1^: relative value of total distance, PL.min^−1^: relative value of player load, RHIE.min^−1^: relative value of repetitive high-intensity exercise.

As for SSGs with different rules, overall, the relative workload of TC was the highest. Common to all SSG models, m.min^−1^ for forwards and backs were all more required than OGs (Figures 5A, B, *p* < 0.05, η_p_^2^ = 0.57). Only the forwards in the 7 vs. 4 model had a lower PL.min^−1^ than the OG (Figure 5C, *p* < 0.05, ES = 0.03), the rest of the models met the game requirements in backs (Figure 5D, *p* < 0.05, η_p_^2^ = 0.14). For both forwards and backs, OG had a higher RHIE.min^−1^ requirement than the JT model (Figures 5E, F, *p* < 0.05, ES = 0.29), and that value of backs was also lower than OG in the TRL model (Figure 5F, *p* < 0.05, ES = 0.06).

## Discussion

This study conducted a comprehensive workload analysis of different SSG models and their effects on male professional RU players whilst taking playing positions in consideration. Specifically, the research examined SSG workload with different numbers of players and pitch sizes on strength and endurance training days. It also investigated the differences in workload between SSGs using four different rules. In addition, the study checked whether all SSGs complied with OG requirements. The primary findings of this study provide evidence that running-related metrics of SSGs were higher on endurance days, whereas mechanical workload was higher on strength training days. When comparing the effect of different numbers of players on SSGs, the TD of the players was greatest in the 7 vs. 7 S-SSG model. Furthermore, SSG models with four rules show that forwards usually have lower workload values than backs with respect to position. Among these SSG models, acceleration and deceleration related metrics were highest in the 7 vs. 4 model. The V_max_, HSR, VSHR, and RHIE were all observed with JT model. Players had the highest TD and PL during TC model. Moreover, the relative TD of both S-SSG and E-SSG models, as well as the SSGs set by four rules, fully meet or significantly exceeded the OGs requirements, thereby confirming our initial working hypothesis.

### Data variabilities

One of the objectives of this study throughout the data evaluation process was to investigate the repeatability of the data due to the limited sample size of the data from S-SSG and E-SSG models. TD and mechanical metrics had low variability, suggesting that coaches can confidently establish similar distance and acceleration-deceleration training programs for SSGs. However, the reproducibility of running performance cannot be guaranteed. Given this, coaches must be aware that high- and very high-speed running should be suitably formed in dedicated or specialized training sessions, or that conditions need to be modified to make these metrics replicable (Clemente et al., 2019). For instance, when comparing players within similar positional groups, the significant variability issue should be taken into consideration. We encourage future research to further refine the categorization of players within the forward and back positions (e.g., “front row,” “inside backs,” etc.) in training interventions (Quarrie et al., 2013; McLaren et al., 2016).

### Comparison between forwards and backs

There has always been a difference in the workload of forwards and backs (Cahill et al., 2013), so the data should be processed in accordance with the characteristics of each position. Typically, when conducting rugby training or games, backs are subject to higher external workload than forwards (McLellan et al., 2011; Hu et al., 2023). The same tendency was observed in this study. This is due to the fact that players in different positions were required to comply with the characteristics of the game in daily training. Backs spend more time performing strenuous running activities, acceleration and deceleration movements because of the nature of their role which requires them to cover longer distances to reach their opponents. Additionally, they must sprint while carrying out additional duties like kick chases and returns. These factors inevitably lengthen their running distance (King et al., 2009). Forwards should prioritize training acceleration due to the comparatively shorter average distance covered in high-intensity running reported for this positional group (Austin and Kelly, 2013).

### Effects of modifying pitch size and player numbers

Manipulating the number of players and the pitch sizes in SSGs affects the skills and performance of RU players (Fleay et al., 2018; Zanin et al., 2021). Foster et al. (2010) compared the effects of 4 vs. 4 and 6 vs. 6 and three pitch sizes (15 × 25 m, 20 × 30 m, and 25 × 30 m) on the HR responses of rugby league players. Their findings revealed that a suitable method for raising SSG intensity was to reduce the number of players while maintaining the same pitch size. Research by Kennett et al. (2012b) also supported this, claiming that the time-motion demands were higher in smaller SSG models (4 vs. 4). In our study, the workload of players during 7 vs. 7 was greater than during 8 vs. 8 when pitch size was set at 2,500 m^2^. Our study also showed that a combination of 4 vs. 4 and 660 m^2^ had most demands in accelerations and decelerations, hence the findings were consistent. However, among elite youth junior rugby league players, pitch size had no effect on their physical demands (Dudley et al., 2023). Moreover, Hill-Haas et al. (2010) demonstrated that adding a floater (3 vs. 3 + 1 floater) during SSGs could offer a training stimulus that was more favorable to aerobic adaptation. However, the presence of a floater in the 4 vs. 4 model did not play a positive role in this study. When programming SSGs to address different training objectives, these findings may have practical implications. For instance, on endurance training days, specifying larger pitch dimensions or a greater number of players (7 vs. 7, 2500 m^2^, 178.57 m^2^ per player) can maximize distance and high-speed running training. Conversely, on speed training days, prescribing smaller pitch dimensions or fewer players (4 vs. 4, 660 m^2^, 82.5 m^2^ per player) can achieve optimal acceleration training. Therefore, these recommendations can assist coaches in adjusting SSGs more purposefully to meet various training needs (Tee et al., 2018).

### Effects of modifying game rules

An interesting result of this study is that the 7 vs. 4 model resulted in the highest workload on the players during medium-to-hard-acceleration movements because of the smallest pitch size used. This increased workload can be attributed to the players’ necessity to change direction quickly and perform acceleration and deceleration movements over shorter distances. From a practical standpoint, it informs the implementation of SSGs designed to expose players to the strength demands such as explosiveness, agility and acceleration of games (Baker and Newton, 2008). Besides, players can obtain more HSR training by practicing the JT model. TRL model with its primary focus on aerobic training, was designed to be more moderate in workload. As a result, given its avoidance of extreme high-intensity training, it was often used at the beginning of the training week to avoid excessive workload and aligns more closely with the demands of RU. The objective of the TC model was to enhance stamina and power capabilities through a combination of aerobic and speed training; hence its intensity was likewise quite modest. Consequently, SSGs can be chosen or altered based on the training objective.

### Comparison between SSGs and OGs

Another objective of this study was to use several relative metrics to compare the movement patterns of SSGs and OGs in RU, indicating that SSGs can partly meet game expectations. Similar studies have been reported in other team sports. For instance, field hockey has shown that acceleration counts were higher in SSGs than in games when normalized to 70 min of game time (Gabbett, 2010). Moreover, all SSG models (5 min; 2 vs. 2, 3 vs*.* 3, and 4 vs*.* 4; on pitches of 30 × 20, 35 × 25, and 40 × 30 m) in field hockey had higher mean acceleration, deceleration, and change-of-direction events than the maximum average over a 5-min period of game. However, the average speed was significantly lower during SSG models (Duthie et al., 2022). In football research, only 4 vs. 4 SSG showed the same PL and accelerations as OGs, whose values were obtained by calculating the rolling average over a 5-minute period and selecting the highest ones (Dalen et al., 2021). Additionally, Casamichana et al. (2012) found that VHSR and all RHIE variable metrics were observed to be higher in friendly games compared to SSGs. By contrast, only SSGs exhibited higher values compared to friendly games in VHSR and RHIE/min. In this study, when the SSG models did not satisfy the OGs, it was mainly due to differences in model functionality. For example, the TRL model was primarily used to recover or restart training due to the lower workload it encompasses. The differences in these results are limited by the different sports and metrics, necessitating additional research on RU (e.g., development and modification of SSG models., integrating video analysis or physiological indicators into research).

### Limitations

There have been a few limitations to this study. Considering the training demands of players’ actual preparation for matches, we did not conduct a controlled experiment. When designing the protocol, we considered controlling the number of players to change the pitch size, as well as controlling the pitch size to change the number of players, but then this experimental design would be very long and take a lot of time. This would not be supported as it would put a lot of extra burden on the professional players (the same situation for pre-post performance tests on the S-SSG models). In addition, most of the SSGs research in rugby is controlling the pitch size to change the number of players or controlling the number of players to change the pitch size (Halouani et al., 2014). This study could be a new attempt at the format of SSGs for the purpose of using SSGs on strength training days and endurance training days. In our study, no improvement in performance was found after training, and also extremely significant differences between SSGs and OGs warrants consideration of overtraining (Gómez-Carmona et al., 2018), implying that future studies should take this into account. Furthermore, due to the training schedules and testing feasibility for professional players, we had no chance of obtaining internal workload data (such as sRPE, blood lactate, and HR values). Moreover, since forwards tend to have heavier physical tasks as they engage in mauls, rucks, and scrums (Duthie et al., 2003b), only the high-intensity work of backs can be reflected in the GPS data, we should have assessed metrics that are more reflective of the workload on the forwards (such as sRPE and sprint momentum). Finally, considering all of the players were from the same team, it is uncertain whether the results can be extrapolated to other teams and other playing levels.

## Conclusion

This study served as a practical reference for coaches to develop training regimens for SSGs and provided evidence to quantify the differences in workload between SSGs and OGs for male professional RU players. SSGs usually showed higher requirements in external workload metrics and can therefore cover the workload of games. In the context of consistent training strategies, coaches can optimize different SSGs to meet physical and game goals. To guarantee that players will receive adequate stimulation for training, it is also necessary to maintain a suitable number of players, maintain the proper size of the field, and select appropriate rules according to the characteristics of the position. Further studies utilizing the methods described here are warranted due to the interest in this topic for rugby training.

## Data Availability

The raw data supporting the conclusion of this article will be made available by the authors, without undue reservation.
